# Potential drivers of HIV acquisition in African-American women related to mass incarceration: an agent-based modelling study

**DOI:** 10.1186/s12889-018-6304-x

**Published:** 2018-12-18

**Authors:** Joëlla W. Adams, Mark N. Lurie, Maximilian R. F. King, Kathleen A. Brady, Sandro Galea, Samuel R. Friedman, Maria R. Khan, Brandon D. L. Marshall

**Affiliations:** 1 Brown University School of Public Health, 121 South Main Street, Providence, RI 02912 USA; 2 Philadelphia Department of Public Health, AIDS Activities Coordinating Office, Philadelphia, PA USA; 3 Boston University School of Public Health, Boston, MA USA; 4 National Development and Research Institutes, New York City, NY USA; 5 Division of Comparative Effectiveness and Decision Science, Department of Population Health, New York University, New York City, NY USA

**Keywords:** Systems analysis, HIV, Prisons, Inmate, Sexual behavior, African Americans

## Abstract

**Background:**

The United States has the highest incarceration rate in the world. Incarceration can increase HIV risk behaviors for individuals involved with the criminal justice system and may be a driver of HIV acquisition within the community.

**Methods:**

We used an agent-based model to simulate HIV transmission in a sexual-contact network representing heterosexual African American men and women in Philadelphia to identify factors influencing the impact of male mass incarceration on HIV acquisition in women. The model was calibrated using surveillance data and assumed incarceration increased the number of sexual contacts and decreased HIV care engagement for men post-release. Incarceration of a partner increased the number of sexual contacts for women. We compared a counterfactual scenario with no incarceration to scenarios varying key parameters to determine what factors drove HIV acquisition in women.

**Results:**

Setting the duration of male high-risk sexual behavior to two years post-release increased the number of HIV transmissions to women by more than 20%. Decreasing post-release HIV care engagement and increasing HIV acquisition risk attributable to sexually transmitted infections (STIs) also increased the number of HIV transmissions to women. Changing the duration of risk behavior for women, the proportion of women engaging in higher risk behavior, and the relative risk of incarceration for HIV-infected men had minimal impact.

**Conclusion:**

The mass incarceration of African American men can increase HIV acquisition in African American women on a population-level through factors including post-release high-risk behaviors, disruption of HIV care engagement among formerly incarcerated men, and increased STI prevalence. These findings suggest that the most influential points of intervention may be programs seeking to reduce male risk behaviors and promote HIV care engagement post-release, as well as STI testing and treatment programs for recently incarcerated men, as well as women with incarcerated partners.

**Electronic supplementary material:**

The online version of this article (10.1186/s12889-018-6304-x) contains supplementary material, which is available to authorized users.

## Background

The United States is home to less than 5 % of the world’s population yet imprisons over a fifth of the world’s prisoners [1]. As of 2015, over 2.2 million Americans were held in penal institutions, either awaiting trial or serving a sentence [1]. As a result, incarceration has become a “normal life event” for many men and women in the US, particularly within communities of color. The “war on drugs”, racial biases in arrests and sentencing, and other structural factors have led to marked racial disparities in incarceration [2–4]. By the age of 34, an African American male with less than a high school education has a 69% probability of having spent time in prison or jail [5].

The mass incarceration of African American men is hypothesized to play an important role in racial HIV disparities [6–10]. Heterosexual African Americans with a history of incarceration are six times more likely to be HIV-infected compared to those with no incarceration history, and numerous observational studies suggest a history of incarceration is a strong and consistent risk factor for high-risk sexual behaviors post-release [6, 11–17]. A study in Baltimore found that 28% of men with a recent incarceration event had five or more sexual contacts in the past year and 32% reported partner concurrency (defined as partnerships that overlap in time) [6]. Additionally, incarceration can disrupt HIV care engagement. Recently incarcerated HIV-infected men are more likely to disengage from HIV care and experience viral rebound post-release, which in turn increases HIV-related morbidity and the likelihood of HIV transmission to their sexual and drug-using partners [18–24]. Consequently, the mass incarceration of men may be a major driver of HIV acquisition for women.

Mass incarceration disproportionately affects African American men in urban, low-income communities whose partners are often already at high risk for sexual HIV transmission. Up to 80% of inmates are married or in committed relationships at the time of incarceration [14, 25]. It is estimated that over half of these relationships end during incarceration; thus, incarceration results in the dissolution of sexual partnerships and the disruption of social networks, which can result in negative behavioral outcomes that increase HIV risk [10, 26]. Previous studies have reported an increased risk of sexually transmitted infections (STIs), a greater likelihood of having five or more partners in the past year, and an increased prevalence of partner concurrency among women with a recently incarcerated male partner [6, 27]. However, it is not known what factors related to the mass incarceration of African American men affect HIV transmission and prevalence among women at the population level. The current analysis used an agent-based model (ABM) to determine what factors modify the impact of the mass incarceration of African American men on HIV acquisition in African American women, using the city of Philadelphia as a case study. Philadelphia was selected as the setting for the model as the city has the highest per capita incarceration rate of the 10 largest U.S. cities, with 2% of African American men behind bars [28].

ABMs have several distinct advantages compared to other methods for this research question [29, 30]. Agent-based modelling is of particular utility when interference (i.e., the outcome of one individual influences the exposure status of others) dominates the behavior of the system, and thus influences exposure-disease relationships [31]. ABMs allow us to model changes in risk behavior (e.g., an increase in sexual partners) that often occur after the incarceration of an individual in that agent’s sexual network. Additionally, ABMs can be used to examine the effect of multiple exposures that interact in dynamic ways to affect population-level health outcomes [31]. ABMs have been used to estimate the effect of intervention strategies in preventing HIV transmission within networks of people who inject drugs, understand the contribution of acute HIV infection to community HIV incidence, and detail HIV transmission dynamics [32–36]. Through explicitly simulating both the individual behavioral and network-level effects of incarceration, use of an ABM allows us to address questions of HIV acquisition attributable to mass incarceration.

## Methods

We used the Treatment of Infection and Transmission in Agent-Based Networks (TITAN) model, a dynamic model of HIV transmission. The TITAN model is an ideal platform for this analysis as the model has successfully been used to explore how racial inequities in pre-exposure prophylaxis (PrEP) programs, treatment as prevention efforts among people who inject drugs, and other complex processes impact HIV transmission within a mature epidemic setting [37–39]. The TITAN model is parameterized from peer-reviewed literature, surveillance data, and other sources and then calibrated to reflect the real-world trends in HIV transmission. This analysis simulated the movement of men in and out of prison or jail to understand the interaction between incarceration and HIV acquisition in African American women. The TITAN model was run with discrete monthly time-steps and an open population. At each time step, sexual relationships or “links” between agents are formed, retained, or broken, thus representing the evolution of a population-based sexual HIV transmission network. Full details for this model, including model parameterization and technical specifications, are provided in the supplemental material.

### Model population

The agent-based model used for this analysis generated a virtual population representing the total population of heterosexual African American men and women (*n* = 440,000) living in Philadelphia from 2005 to 2015. Although the model initializes the network in 2005, the first three years of model simulation (i.e., the burn-in period) are not included in the main results, as this period was necessary in order to reach a steady-state and accurately reflect historical trends in empirical data for the *status quo* model.

Each individual agent had both fixed (e.g., gender) and dynamic (e.g., HIV infection status, incarceration status) characteristics. HIV-infected agents had attributes (e.g., HIV diagnosis status) that varied over time according to care engagement. Sexual networks were assumed to be exclusively heterosexual and relationships were defined as either main (≥1 month in duration) or casual (≤1 month in duration). Agents had differing probabilities of mortality depending on gender, HIV infection status, and HIV disease stage. An overview of model parameter and processes is presented in Table 1. Parameters and data sources used to define agent characteristics are fully described in Additional file 1: Tables S1-S7 within the supplement.

**Table 1:** Overview of model parameters and processes

Processes	Description
Demography	
Gender	Seeded at baseline based on gender distribution reported in the 2000 U.S. Census.
Mortality	Implemented based on HIV disease stage, gender, and use of antiretroviral therapy.
Sexual Behavior and Sexual Network	
Sexual partner preference	Female agents can only partner with male agents and vice versa. Seeded stochastically at baseline based on empirical studies.
Condom use	Probability of condom use is based on relationship duration (< or ≥ 1 month) and HIV diagnosis status.
**Partner acquisition rate**	**Agents assigned a personal annual mean number of partners, which is allowed to vary stochastically year-to-year.**
Sex frequency	Agents stochastically assigned a desired number of sex acts per partner per year. At the partnership level the resulting number of sex acts represents a compromise between the two partners.
**Relationship length**	**Varies stochastically based on empirical data on mean and median relationship lengths. Agents have a 50% likelihood of relationship dissolution during incarceration.**
Incarceration	
Incarceration rate	Derived from 2005 data from the Philadelphia Commission on Sentencing for African American men, held constant through model run. Varied by type of correctional facility (jail vs. prison) and recidivism status (prior offense vs. first offence).
Sentence length	Derived from 2005 data from the Philadelphia Commission on Sentencing for African American men, held constant through model run. Varied by type of correctional facility (jail vs. prison).
HIV/AIDS	
Initial HIV prevalence	Based on HIV surveillance data for African American men and women in Philadelphia.
**Testing**	**Agents test stochastically throughout the year with differing probabilities based on gender. Diagnosed agents are less likely to transmit to HIV-negative partners.**
**Viral suppression**	**Only HIV-diagnosed agents are eligible for viral suppression. Virally suppressed agents are less likely to transmit to HIV-negative partners.**
**HAART discontinuation**	**Only HIV-diagnosed agents are eligible to initiate and discontinue HAART. Annual probability of discontinuation differs by gender and incarceration status.**
**Transmissibility**	**Based on STI status, HIV diagnosis status, HAART adherence, and disease stage.**

*Abbreviations: HAART* highly active antiretroviral therapy, *STI* sexually transmitted infection

Bolded parameters are those impacted by incarceration or partner incarceration in order to simulate a “high-risk” period

### HIV prevalence, screening and treatment

Data from the AIDS Activities Coordinating Office (AACO), the HIV surveillance unit in the Philadelphia Department of Public Health, were used to parameterize and calibrate trends in HIV prevalence and incidence. At model initialization (representing the year 2005), the HIV prevalence was 1.58% for African American men and 1.18% for African American women. Following historical trends, the number of HIV-infected individuals on highly active antiretroviral therapy (HAART) and achieving viral suppression increased over time. Selected parameters were held constant throughout model simulations, including the proportion of HIV-infected individuals who were diagnosed (75%), the annual probability of HAART discontinuation within the general community (42% for men and 52% for women), and the monthly probability of HIV testing for the community (3.4% for men and 3.9% for women) based on published data (see Additional file 1: Table S3 within the supplement).

### Incarceration

In November 2005, 2.7–2.8% of Philadelphia’s male African American population over the age of 18 was currently incarcerated [28]. In our model, incarceration was defined as being held in a prison or any other kind of detention facility for at least one month or longer. At model initialization, 2.74% of the male agents were incarcerated. Detailed information using 2006 data from the Philadelphia Commission on Sentencing on sentence lengths and incarceration rates for African American men in Philadelphia were used to parameterize the model [40]. Rates of incarceration and sentence lengths were reported by type of correctional facility (jail vs. prison) and recidivism status (first-time offender vs. prior incarceration). For example, the annual rate of incarceration in prison for African American men with a prior record was 251 per 100,000 with an average minimum sentence of 45.6 months. These rates and sentence lengths were held constant through model runs.

Based on previously published research on HIV testing within Philadelphia correctional facilities, we assumed that 69% of agents were tested for HIV at intake [41]. Consistent with available data, 40% of diagnosed HIV-infected men were assumed to achieve viral suppression during incarceration [23]. Individuals experiencing incarceration have multiple barriers to achieving viral suppression while incarcerated including the lack of universal HIV testing at intake, limited infrastructure or staffing (e.g., infectious disease physician), as well as challenges with HAART adherence [23, 41]. Since we were interested in examining the effect of male incarceration on women’s HIV acquisition, only male agents were eligible to experience incarceration.

Within the model, risk behaviors were parameterized using observational studies on the impact of incarceration and partner incarceration [14, 15, 23, 42, 43]. During incarceration, the agent ceases all sexual contact; however, relationships can be maintained so that a proportion of relationships last through incarceration and sexual activity resumes post-release. The model assumed that the probability of relationship dissolution was higher if the male agent entered jail or prison, resulting in increased relationship turnover among those incarcerated and their partners [26].

Women were eligible to experience increased risk behavior only if the incarcerated partner was a main partner. Women had a higher probability of entering the “high-risk group” if a relationship dissolved during incarceration. High-risk behavior for women consisted of a higher mean number of sexual partners and was initiated immediately upon the partner’s incarceration and continued throughout the partner’s incarceration or until six months following the relationship dissolution [42]. For the six months following release from either prison or jail, all men entered a period of high-risk behavior with an increased number of sexual partners from a median of 3 (interquartile range: 1,7) partners per year to an average of 1.8 partners per month [14].

**Fig. 1:**
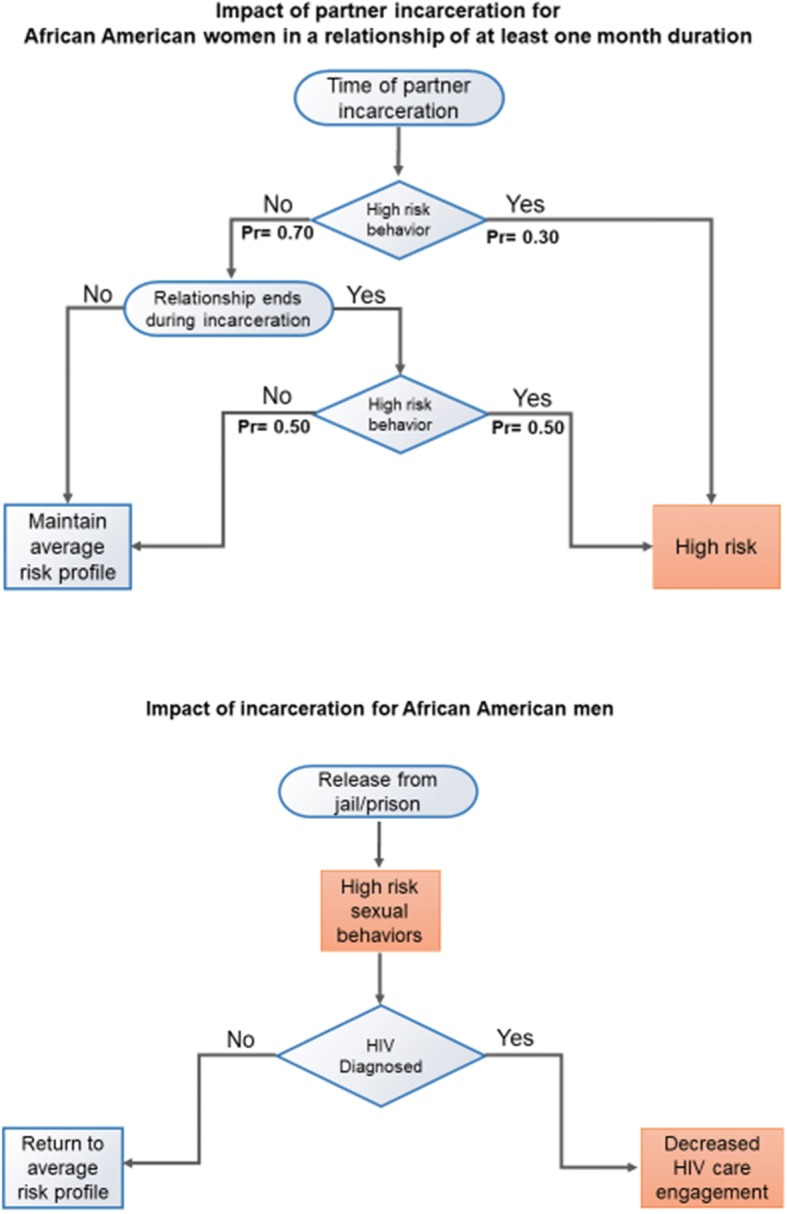
The impact of incarceration and partner incarceration for agents within the ABM model

Incarceration also impacted HIV care engagement. A recent systematic review of national data found that 51% of HIV diagnosed prisoners are on HAART while incarcerated, but only 29% are on HAART after release [23]. Therefore, within our model, only half of the HIV-infected men who were virally suppressed during incarceration maintained viral suppression by six months post-release [23]. In summary, incarceration or partner incarceration was modeled to directly increase the mean number of sexual partners, decrease the probability of maintaining viral suppression for male HIV-infected agents, and increase the probability of relationship dissolution. Increased rates of partner concurrency and relationship turnover emerged as a result of these changed behaviors rather than as a result of pre-programmed parameters. Figure 1 reviews the impact of male incarceration within the model.

### Model scenarios

A *status quo* scenario was calibrated using surveillance data on HIV prevalence and incidence rates for the study period (2005–2015). Six scenarios varying key parameters hypothesized to influence the impact of mass incarceration were then performed. Specifically, we varied the duration of high-risk behavior for men post-release (3 months, 24 months), duration of high-risk behavior for women (3 months, 24 months), the proportion of women engaging in high-risk behavior (0, 100%), HAART coverage for HIV-infected men at six months post-release (0, 100%), the relative risk of incarceration for HIV-infected men compared to HIV-uninfected men (2, 5), and doubled the risk of HIV transmission per unprotected vaginal sex act to account for the increased probability of a current STI among high-risk agents (for high-risk period only, for the remainder of the model run). In order to isolate the effect of the selected parameter, we held values for the other key parameters constant at a referent value. Each scenario was run 100 times with ¼ the total population size (*n* = 110,000) using Markov Chain Monte Carlo simulations; results were then scaled to reflect the target population (*n* = 440,000).

For each scenario, we projected the HIV incidence and prevalence rates and total number of transmission events for African American women over the study period. Model scenarios were compared to a counterfactual scenario where no agent experienced incarceration by calculating the mean difference in the number of new HIV infections among African American women.

## Results

The model was successfully calibrated to reflect HIV prevalence and incidence trends for African American men and women living in Philadelphia from 2005 to 2015. Over the study period, the average HIV prevalence was 2397 per 100,000 for men and 1857 per 100,000 for women, and the average annual HIV incidence was 75 per 100,000 for men and 64 per 100,000 for women. Over the entire study period, including the three-year run-in period, 9.6% (*n* = 17,117) of men and 8.3% (*n* = 380) of HIV-infected men experienced at least one episode of incarceration. On average, 8.1% (*n* = 21,096) of women experienced a period of high-risk behavior related to a partner’s incarceration. There were 1641 (1421-1881) transmission events among women over the 10-year period in the *status quo* scenario with referent values for key parameters. We then ran a counterfactual scenario with no incarceration (i.e., incarceration rate was set to 0). In the absence of incarceration, there were 1632 (95% SI: 1427-1861) HIV transmissions among women over ten years. The number and percent difference in cumulative HIV infections among women for scenarios varying key parameters compared to the scenario without incarceration are presented in Table 2.

**Table 2:** Average number of cumulative new HIV infections among African American women over 10-year period and mean difference in the number of transmissions by scenario

Scenarios varying key parameters related to mass incarceration	Incarceration (*status quo)* scenario (N, 95% Simulation Interval [SI])	Mean difference (*status quo*- no incarceration scenario)^a^ N, %
*Male duration of high risk behavior post-release*		
3 months	1607 (1408-1804)	− 25 (− 1.6%)
6 months (referent)	1641 (1421-1881)	9 (0.5%)
24 months	2081 (1751-2429)	449 (21.6%)
*Female duration of high risk behavior*		
3 months	1639 (1394-1874)	7 (0.4%)
6 months (referent)	1641 (1421-1881)	9 (0.5%)
24 months	1673 (1426-1879)	41 (2.5%)
*Female proportion engaging in high risk behavior*		
0%	1652 (1426-1923)	20 (1.2%)
40–60% (referent)	1641 (1421-1881)	9 (0.5%)
100%	1652 (1452-1879)	20 (1.2%)
*ART coverage post-release*		
0%	1767 (1518-1980)	135 (7.6%)
21% (referent)	1641 (1421-1881)	9 (0.5%)
100%	1613 (1386-1808)	− 19 (− 1.2%)
*Relative risk (RR) of incarceration for HIV+ men* vs. *HIV-uninfected men*		
RR = 1 (referent)	1641 (1421-1881)	9 (0.5%)
RR = 2	1618 (1430-1848)	− 14 (− 0.9%)
RR = 5	1609 (1399-1822)	− 23 (− 1.4%)
*Doubled HIV acquisition risk per unprotected sex act due to increased STI risk among high-risk agents*		
Not implemented (referent)	1641 (1421-1881)	9 (0.5%)
High-risk period only	1658 (1439-1892)	26 (1.6%)
Remainder of model run	1816 (1619-2002)	184 (10.1%)

^a^Scenario with no incarceration had 1632 (1427-1861) HIV transmissions

**Fig. 2:**
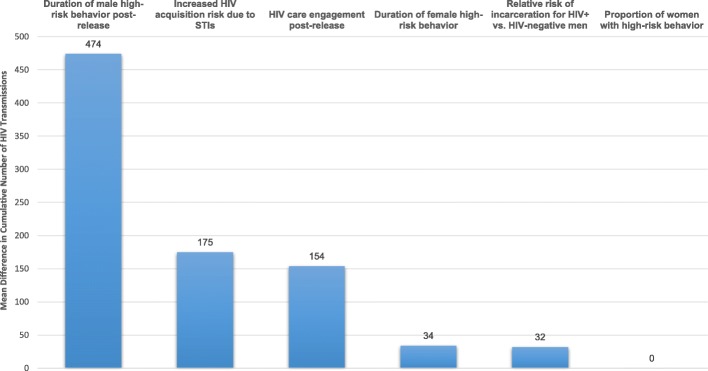
HIV transmissions attributable to changing selected parameter from upper to lower bound value^a^. ^a^The y-axis represents the number of HIV transmissions attributable to changing the selected parameter from lower to upper bound value. Calculated as the absolute difference in the mean number of HIV transmissions using values reported in Table 1. For the male duration of high risk behavior parameter this is equivalent to 449-(-25) =474

Increasing the duration of male high-risk behavior post-release had the greatest impact on HIV acquisition in women (see Fig. 2**)**. If risk behavior lasted for 24 months, there were 449 additional HIV infections to women over the study period compared to the no incarceration scenario. Decreasing HAART coverage to 0% for men post-release (i.e., no men were virally suppressed by six months post-release), resulted in 135 additional infections. Doubling the HIV transmission risk per unprotected vaginal sex act for high-risk men and women to account for the increased probability of an STI increased the number of HIV transmissions. When the increased HIV transmission risk was limited to the high-risk period, there were 26 additional HIV infections. If high-risk agents were assumed to have an increased HIV transmission risk for the remainder of the study period, there were 184 additional HIV infections. Scenarios varying the duration of female risk behavior, the proportion of women engaging in high-risk behavior, and the relative risk of incarceration for HIV-infected men compared to uninfected men had a minimal impact on HIV transmissions.

## Discussion

Using Philadelphia as a case study, we found that the mass incarceration of African American men can substantially increase the number of HIV transmissions to African American women within the community. Factors including post-release high-risk behaviors, disruption of HIV care among formerly incarcerated men, and increased STI prevalence significantly modified the impact of incarceration on HIV acquisition in women. These factors have the potential to increase the number of HIV transmissions among women attributable to mass incarceration and may represent targets for intervention efforts.

To our knowledge, this is the first study to evaluate the impact of behavioral, network, and care engagement processes associated with mass male incarceration on HIV acquisition in women. The impact of incarceration varied depending on several factors. Assuming that high-risk behavior of men post-release lasted two years resulted in a greater than 20% increase in the number of HIV transmissions among women compared to the no incarceration scenario. Limited empirical data exists for parameterization of the duration (e.g., 6 vs. 24 months) of increased sexual risk behaviors for men after incarceration. The majority of existing studies have focused on recently released men, typically interviewing men within the first year post-release and finding increased sexual risk behaviors [13, 14, 43]. While our model assumed the duration of high-risk behavior was limited to no more than the first two years post-release, it is plausible that men with a history of incarceration become more likely to engage in HIV risk behaviors for years, or even for the rest of their lives [12]. Consequently, we may be underestimating the potential impact of mass incarceration. Longitudinal studies that follow men post-release for several years are needed in order to better understand the long-term impact of incarceration on sexual risk behavior.

Women with an incarcerated partner in the past year are over twice as likely to have a current STI compared to women without a recently incarcerated partner [6]. STI prevalence is also higher among men with a recent incarceration compared to those without a history of incarceration [6, 44, 45]. A current STI increases the likelihood of HIV acquisition: ranging from a two-fold increase with bacterial vaginosis to a seven-fold increase with gonorrhea [46]. The same processes through which incarceration contributes to HIV incidence (e.g., partner concurrency, increased partners) are also likely to have the same effect upon STI incidence rates. Therefore, we doubled the probability of HIV transmission per unprotected vaginal sex act for high-risk individuals (men and women) to account for this increased likelihood of a current STI. This led to significant differences in the number of HIV transmissions between the no incarceration and the other scenarios accounting for this increased HIV acquisition risk. These findings suggest that STIs are an important and modifiable factor related to the impact of incarceration on HIV acquisition.

Since the U.S. Supreme Court ruling in 1976 (*Estelle v. Gamble*) established the constitutional right to basic health care in correctional facilities, facilities have been mandated to provide prison health services for acute and chronic conditions [47]. However, a systematic review using national data estimated that only 40% of individuals living with HIV achieve viral suppression while incarcerated [23]. Opt-out HIV testing upon intake at a correctional facility has been shown to be both feasible and acceptable and represents the first step to ensuring increased HIV care engagement for individuals experiencing incarceration [48]. Providing support for HIV care including access to HIV providers, adherence counselors, and other services while individuals are incarcerated can improve care engagement during and after incarceration. In addition, there is a need for programs to reduce the risk of HIV transmission (e.g., condom distribution) during incarceration. Even in the presence of these barriers, numerous studies have found that HIV-infected men have improved HIV care engagement and viral suppression while incarcerated compared to engagement when in the community [4, 21, 22, 49, 50]. Substantial barriers to HIV care engagement post-release include both structural and socioeconomic factors [51]. Following release from a correctional facility, many individuals return to the community without HAART medication to take home or a follow-up appointment with an HIV care provider [18, 52]. Many men and women lack health insurance or are required to reapply for health insurance post-release [53]. These logistical barriers often lead to treatment interruptions. In addition, significant socioeconomic factors frequently challenge HIV care engagement. Lack of stable housing, poverty, and stigmatization related to both HIV and incarceration decrease the probability of sustained HIV care engagement [47, 52–54]. Mental health and substance use disorders are highly prevalent among HIV-infected prisoners and are associated with decreased ART adherence and increased HIV risk-taking behaviors [55]. Without treatment for mental health and substance use disorders, HIV-infected individuals are more likely to disengage from HIV care and virally rebound [51, 56]. Successfully linking and retaining individuals to HIV care post-release requires addressing the logistical and socioeconomic barriers to care before release as well as the comorbidities that complicate care engagement [51].

Future work with this model will focus on understanding the impact of the HIV care continuum during and after incarceration and PrEP prescription strategies for women impacted by a partner’s incarceration. Within our model, lack of HIV care engagement post-release increased HIV acquisition in women. Efforts to prevent HAART treatment interruptions through interventions such as transitional care coordination, substance use treatment and mental health services, and integrating health service delivery organizations may decrease HIV acquisition in women [22, 57]. Further research is needed to clarify which strategies would be most effective in improving HIV care engagement in order to improve the health of formerly incarcerated persons and prevent HIV transmission to their partners. In addition, there is a need to increase awareness regarding the risk of HIV acquisition associated with a partner’s incarceration for women. Several experts have called for the use of relationship or partner characteristics such as a partner’s concurrency, a partner’s incarceration, or intimate partner violence to help guide HIV prevention efforts [58]. A recent study evaluating an HIV risk index with five hundred African American women in Atlanta found that partner characteristics (including a history of incarceration) better predicted laboratory-confirmed sexually transmitted infection than other indicators, including recent condomless sex [59]. Therefore, we plan to use this model to explore the potential impact of PrEP prescription for women impacted by a partner’s incarceration on HIV transmission dynamics in a future study.

Our study is subject to several limitations. First, ABMs are not able to simulate the breadth of human experiences related to incarceration (e.g., poverty, stigma, drug relapse). Although we provide our virtual population with the ability to replicate a complex set of interactive events, ABMs will never fully capture the complexity of human behavior. In addition, race-specific or Philadelphia-specific data was not available for some parameters, including the duration of high-risk behavior. However, analyses were used to explore the potential impact that changing these parameters had on study findings. Additional HIV risk factors that were not fully captured within our model include assortative mixing (i.e., higher risk individuals are more likely to interact with other high-risk individuals), engagement in other HIV risk behaviors (e.g., injection drug use), or the increased HIV risk among men who have sex with men and women (MSMW) [10]. A modest percentage (approximately 6%) of black men who have sex with women report having a history of sex with men [60]. Therefore, the simulated incarceration effect is conservative to the extent that incarceration increases assortative mixing and the model does not include MSMW or account for other HIV risk behaviors [61].

In addition, we assumed that sexual contact ceased during incarceration despite evidence to the contrary [62]. However, studies have shown the majority of HIV transmission occurs outside of prison facilities and therefore, this assumption is unlikely to significantly impact our results and would have led to the underestimation of impact [63, 64]. Within the model, the probability of being incarcerated did not vary by age as rates are for the general adult population of African American men. Therefore, incarceration was relatively rare compared to what observational studies have shown for subsets of the population (i.e., African American men ages 18–35) [6]. Sexual partner selection was not based on age and therefore, we were not able to take into account the differential prevalence of partner incarceration by age. Therefore, the model underestimated the prevalence of incarceration and partner incarceration for a proportion of the population, and consequently, may have underestimated the impact of incarceration for this subset.

These limitations aside, this study has several strengths. First, with regards to the model calibration, we used validated, population-based surveillance data. Second, many of the model parameters (e.g., likelihood of HIV acquisition per unprotected vaginal sex encounter, mean number of sexual partners) and assumptions have already been tested in previous studies [32–36]. Finally, use of an ABM enabled us to estimate effects that are not possible to measure in a trial or observational study.

## Conclusion

The mass incarceration of African American men can affect HIV acquisition in African American women on the population-level. The duration of male high-risk behavior, disrupted HIV care engagement of men post-release, and increased STI prevalence among those affected by incarceration or partner incarceration potentially drive the effect of mass incarceration on HIV acquisition in women. These findings reinforce the importance of programs seeking to reduce male HIV risk behaviors and promote HIV care engagement post-release as well as STI testing and treatment programs for recently incarcerated men and women with incarcerated partners. These efforts should complement work to reduce overall incarceration rates through criminal justice reform to reduce the number of individuals exposed to the deleterious consequences of incarceration.

## Additional file

Additional file 1: This Supplemental Material includes additional information regarding the structure, parameterization, and results for the agent-based model. The model description follows the ODD (Overview, Design concepts. (PDF 621 kb)

## Abbreviations

*AACO*: AIDS Activities Coordinating Office
*ABM*: Agent-based model
*HAART*: Highly active antiretroviral therapy
*HIV*: Human immunodeficiency virus
*MSMW*: men who have sex with men and women
*PrEP*: Pre-exposure prophylaxis
*STI*: Sexually transmitted infection
*TITAN*: Treatment of Infection and Transmission in Agent-Based Networks
