# Supplementary material for: Potential drivers of HIV acquisition in African-American women related to mass incarceration: an agent-based modelling study
Source: BMC Public Health. 2018 Dec 18;18:1387. doi: 10.1186/s12889-018-6304-x (PMC6299641; doi:10.1186/s12889-018-6304-x)
Supplement: Supplementary file 1 — This Supplemental Material includes additional information regarding the structure, parameterization, and results for the agent-based model. The model description follows the ODD (Overview, Design concepts. (PDF 621 kb) [file 12889_2018_6304_MOESM1_ESM.pdf]

Supplemental Material:

Potential drivers of HIV acquisition in African-American women related to mass incarceration: An agent-based modelling study

Joëlla W. Adams, MPH[1], Mark N. Lurie, PhD[1], Maximilian R.F. King, MSc[1], Kathleen A. Brady, MD[2], Sandro Galea, MD, MPH, DrPH[3], Samuel R. Friedman, PhD[4], Maria R. Khan, PhD[5], Brandon D.L. Marshall, PhD[1]*

[1] Brown University School of Public Health, Providence, Rhode Island, United States of America

[2] AIDS Activities Coordinating Office, Philadelphia Department of Public Health, Philadelphia, Pennsylvania, United States of America

[3] Boston University School of Public Health, Boston, Massachusetts, United States of America

[4] National Development and Research Institutes, New York City, New York, United States of America

[5] Division of Comparative Effectiveness and Decision Science, Department of Population Health, New York University, New York City, New York, United States of America

This Supplemental Material includes additional information regarding the structure, parameterization, and results for the agent-based model. The model description follows the ODD (Overview, Design concepts, Details) protocol for describing individual- and agent-based models.[1,2]

Number of Supplementary Tables/Figures: 8 Tables, 2 Figures

Contents

**i.**	**Study Purpose**	**p. 3**
**ii.**	**State Variables**	**p. 4**
**iii.**	**Process Overview and Sequence**	**p. 6**
**iv.**	**Design**	**p. 7**
**v.**	**Initialization**	**p. 14**
**vi.**	**HIV Disease Progression and Mortality**	**p. 15**
**vii.**	**HIV Transmission**	**p. 17**
**viii.**	**Incarceration**	**p. 19**
**ix.**	***Status Quo* Scenario and Model Scenarios**	**p. 25**
**x.**	**Model Calibration**	**p. 27**
**xi.**	**Technical Details**	**p. 28**
**xii.**	**References**	**p. 29**

Study Purpose

The Treatment of Infection and Transmission in Agent-Based Networks (TITAN) model was developed to simulate HIV transmission dynamics within a mature epidemic setting. The TITAN model has previously been used to estimate the effect of combination intervention strategies in preventing HIV transmission, the impact of acute HIV infection on HIV transmission among people who inject drugs, and to detail HIV transmission dynamics within networks of injection drug use.[3-7] The model simulates HIV transmission, the natural history of HIV disease, as well as HIV screening and treatment. This analysis simulated the movement of men in and out of prison or jail to understand the complex dynamics between incarceration and HIV acquisition in women. The objective of this model was to determine what factors modify the impact of the mass incarceration of African American men on HIV acquisition in African American women.

Agent Entities, State Variables, and Scales

Agent Population

The model consisted of agents representing individuals within the heterosexual African American population of Philadelphia, Pennsylvania aged 18 years or older from 2005-2015. The 2000 U.S. Census reported that there were 262,285 African American women and 191,525 African American men living in Philadelphia aged 18 or older. The percentage of African American men who have sex with men (MSM) in Pennsylvania is estimated to be 4.7%; however, the percentage of MSM is known to be higher within major cities such as Philadelphia.[8] Therefore, we estimated the MSM population to be approximately 7% or 13,407. We subtracted 13,407 from the 2000 Census male population for a final heterosexual male population of 178,118. The final target population

of 440,403 was 40.4% male and 59.6% female. The model allowed for death and the entry of new agents to achieve an open population in steady state.

State Variables

Each individual agent is endowed with state variables or attributes. These include both fixed and dynamic attributes that vary over time (see Table S1). Fixed attributes include gender (male or female) and sexual orientation. For this analysis, agents could only engage in sexual relationships with agents of the opposite sex (i.e., heterosexual).

Dynamic attributes that could change over time were HIV serostatus, HIV diagnosis status, adherence to highly active antiretroviral therapy (HAART), AIDS status, and incarceration status. Each agent was classified as either HIV-infected or HIV-uninfected. HIV-infected agents are either diagnosed or not, were on HAART or not, if on treatment had an adherence level to HAART (0-29%, 30-49%, 50-69%, 70-89%, ≥90%), and AIDS-status (yes/no), dependent on HIV care engagement. HIV disease progression and treatment are described in more detail in the subsection titled “HIV Disease Progression and Treatment”. All male agents could be classified by incarceration status as: a) never incarcerated, b) currently incarcerated, or c) having a history of incarceration. For the purposes of this analysis, female agents were not eligible to experience incarceration within the model. Details on the parameterization of incarceration and partner incarceration are further described within the subsection titled “Incarceration”.

Table S1. Fixed and time variant state variables of agents.

State Variable	Fixed	Time Variant	Data Source
Gender	X		2000 U.S. Census
Sexual Orientation	X		Lieb *et al.*[8]
HIV Serostatus		X	AACO
HIV Diagnosis Status		X	Marks *et al.*[9]
HAART Adherence		X	AACO
AIDS Status		X	AACO
Incarceration Status		X	Goldkamp *et al.*[10], PCS[11], Mauer *et al.*[12]

Abbreviations: AACO- AIDS Activities Coordinating Office within the Philadelphia Department of Public Health; HAART- highly active antiretroviral therapy; PCS- Philadelphia Commission on Sentencing

Time Scale

Within the model, one time step represents one month and simulations were run for 156 months or 13 years. Time was modeled discretely and represented the population of African American men and women living in Philadelphia from 2005-2015. The first three years of model simulation (i.e., the burn-in period) are not included within the reported results, as this period was necessary in order to reach a steady state and accurately reflect historical trends in empirical data for the status quo model. Outcome measures are reported for the last 120 months (i.e., 10 years) of the model runs.

Process Overview and Sequence

The total workflow for the model is diagrammed in **Figure S1**. Additional details are provided below.

Figure S1.

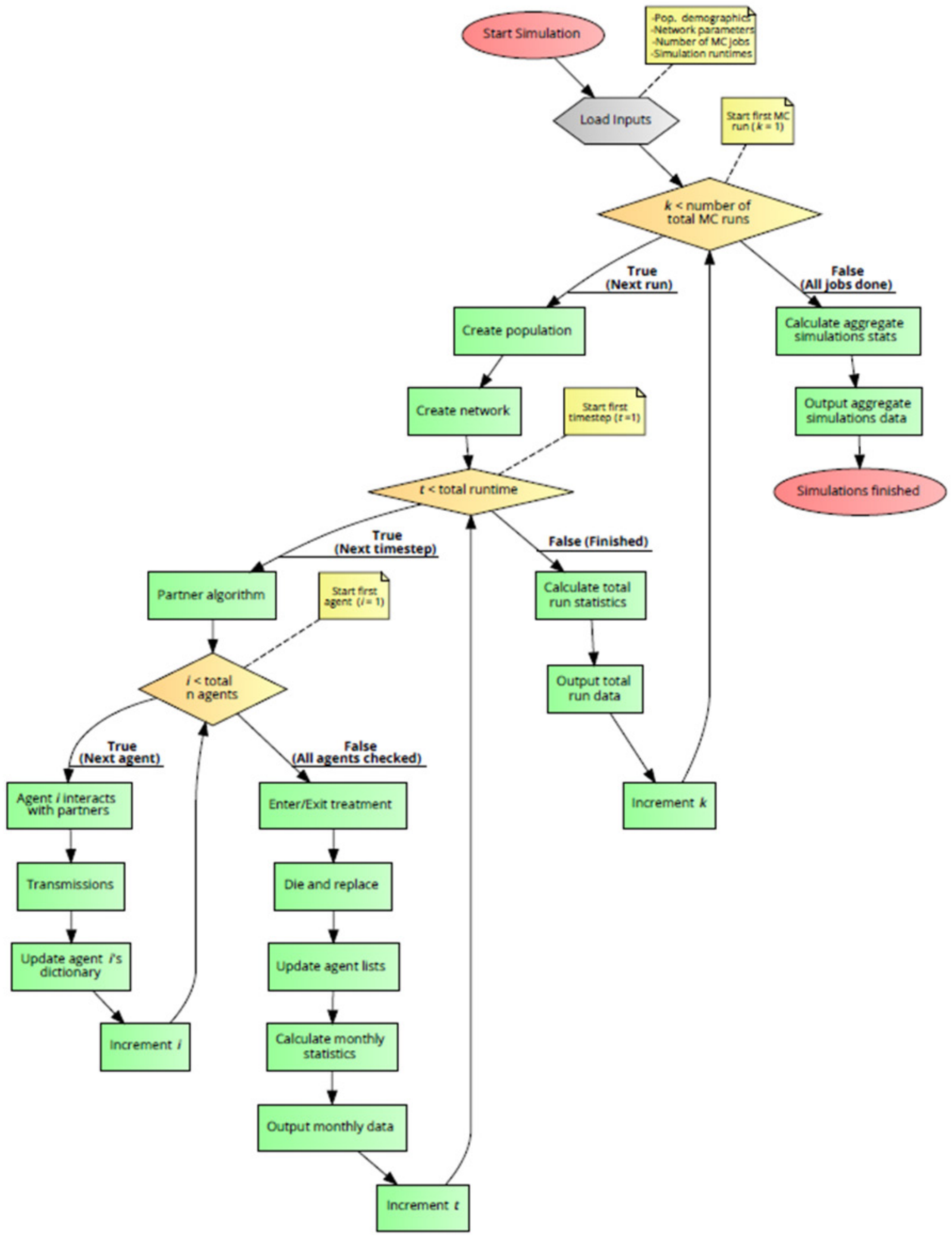

Design

At each time step *t*, agents update their state variables based on pre-programmed rules, interact with other agents, and the environment (i.e., prison/jail versus community). The sequence of workflow for the model is as follows: agents enter partnering algorithm, agents interact with other agents (i.e., sexual contact) within which HIV transmission can occur, state variables related to HIV and high-risk behavior are updated, male agents enter or exit correctional facility, state variables related to incarceration are updated, HIV-undiagnosed agents are eligible for HIV testing, HIV-diagnosed agents are eligible to receive ART, all agents are eligible for death/replacement.

At the beginning of each time step, all male agents not currently incarcerated are eligible to become incarcerated (i.e., enter jail or prison). All actively incarcerated male agents at the beginning of the time step are eligible to be released from jail or prison if their sentence length has been served. Parameterization of incarceration rates and related risk behaviors are described in the subsection titled “Incarceration”. While incarcerated, male agents are not eligible to interact with other agents.

At model initialization and at each time step thereafter, a network is constructed such that each index agent *i* interacts (i.e., has sex with) *j* others in the agent population, where *j* is greater than or equal to zero. Agents are connected to other agents via a casual or main partnership within which unprotected or protected vaginal sexual intercourse can occur. The value of *j* for each agent varies per time step, and is specified by a random variable sampled from a probability distribution function. During transitions between time-steps, agents stochastically form relationship connections. Upon relationship formation, the relationship is given a fixed duration drawn from a distribution. With each time step,

the duration is subtracted until the relationship expires, in which the edge or connection between the two agents is broken and the relationship dissolves.

To construct the network (i.e., form relationships between agents), the program assigns a value $j_{i,t}$ to each index agent i, where $j_{i,t}$ is defined as the number of partnerships with other agents per time step t. The value $j_{i,t}$ is determined by a random sampling procedure from negative binomial (NB) distribution functions, i.e.:

$$J_{i,t} \sim NB(p,r) = \frac{(j_{i,t} + r - 1)!}{(r-1)!\, j_{i,t}!} p^r (1-p)^{j_{i,t}}, \qquad j_{i,t} \in \aleph_0$$

with mean given by:

$$m = \frac{pr}{1-p}$$

for all agents per time step. This method of partner formation means that partners are acquired with probability *p* until *r* suitable partners are found. A previous version of the model used negative binomial distributions to determine partnership formation[7], and the use of negative binomial distributions have been shown by other studies to provide reasonable approximations of real-world partnership networks, in which the variance of the distribution is greater than would be expected assuming constant-rate function (e.g., Poisson).[13]

Probability functions were approximated by the authors based on available data from surveys of sexual partnerships. The majority of these values are extrapolated from studies with published National HIV Behavioral Surveillance (NHBS) data on heterosexuals at increased risk of HIV infection, and calibrated to fit the model's monthly time-steps.[14] The median number of sexual partners per year and interquartile range (IQR) reported for African American men and women in 2013 was 3 (IQR: 1, 7) partners

for men and 2 (IQR: 1,4) partners for women.[15] To avoid overestimating the number of unique partners over consecutive monthly sample drawings, we assigned a partner turnover function that assumes each agent experiences a potential partner turnover event (where they redraw from the partnership distribution) on average once a year. This method ensures that most agents will not form an unrealistic number of unique partnerships over a given time period. The partnering algorithm is diagrammed in **Figure S2**.

Figure S2. Partnering algorithm.

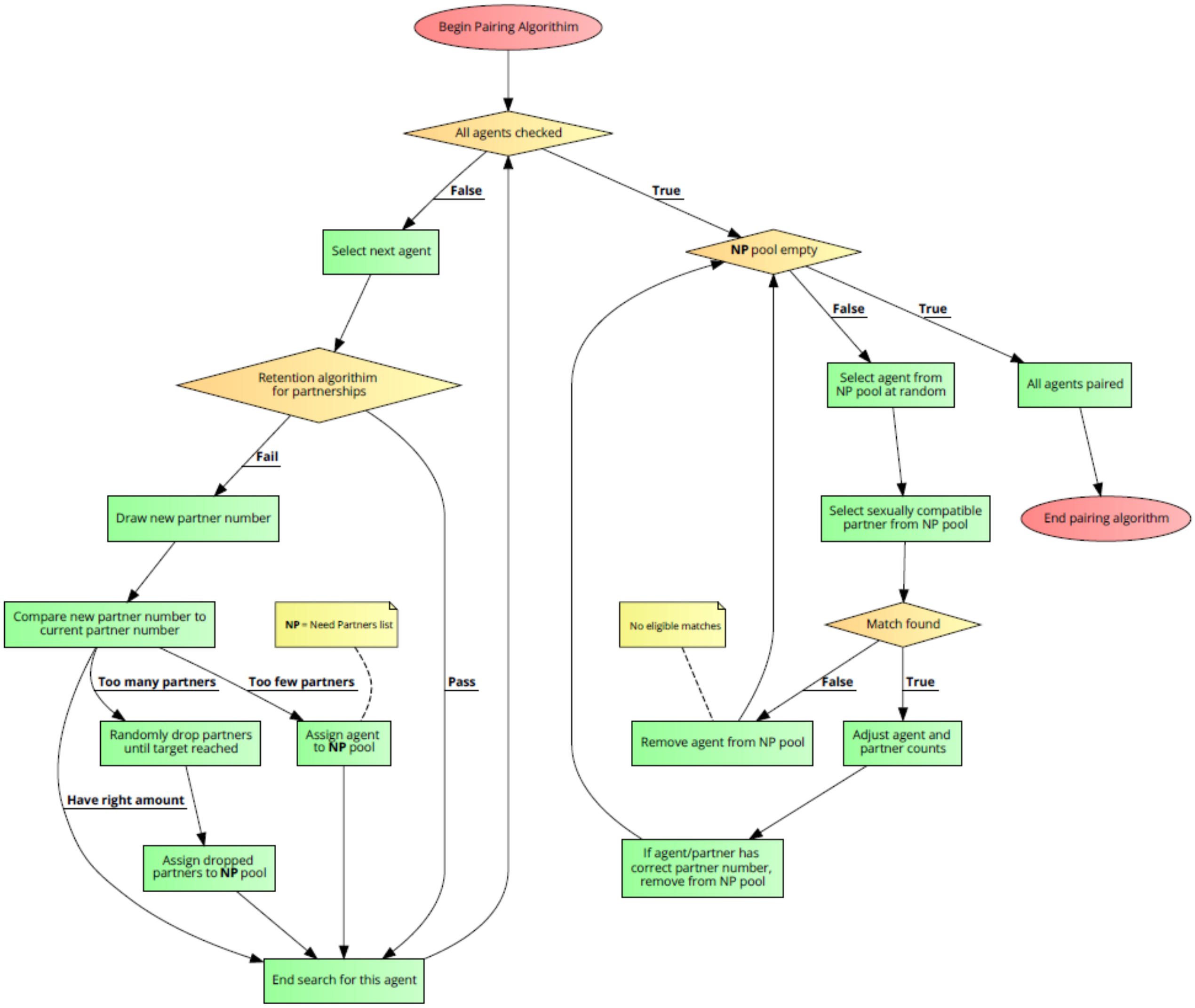

Agents who share a link in the network can engage in vaginal intercourse at each time step. To increase computational efficiency, only sexual activity between serodiscordant agents was simulated. Estimates for non-Hispanic African American men and women within the National Survey of Family Growth (NSFG), a national probability sample, was used to parameterize the mean and median number of times vaginal sexual intercourse was engaged in per time step.[16] The number of vaginal sex acts that a given dyad engages in for a specific time step is determined scholastically using a Poisson-distributed estimate based on these parameters. Relationships (i.e., links) were either classified as main (lasting beyond one time step) or casual (lasting for only one time step). Relationship duration was parameterized using empiric data from the Seattle Sex Partner Survey.[17] The Seattle Sex Partner interviewed 593 heterosexual men and women recruited from two sexually transmitted infection (STI) clinics for a longitudinal study from 1992 to 1995.[17] While a third of the sample was African American, race-specific estimates for the duration of relationships were not reported. Philadelphia-specific estimates for relationship duration and the mean and median number of vaginal sex acts were not available.

The likelihood of unprotected vaginal intercourse was determined stochastically using a per-act probability based on empiric estimates from NHBS survey data on heterosexuals at increased risk of HIV infection.[15] We used national estimates from NHBS, which includes Philadelphia as one of the field sites, for African American men and women interviewed in 2013. Within NHBS, men and women reported having unprotected sex 72% and 73% of the time with causal partners, and 89% and 92% of the time with main partners, respectively.[15] We averaged these probabilities within the model

so that agents within casual relationships (defined as a relationship duration of less than one month or for only one time step) had a 72.5% probability of engaged in unprotected sex while agents within main relationships (defined as a relationship duration one month or more) had a 90.5% probability of engaging in unprotected sex. Sexual behavior parameters and data sources are presented in **Table S2**.

Table S2. Parameter estimates for sexual behavior in general population.

Variable	Base estimate		Data Source
	Male Agents	*Female Agents*	
Sexual partners per year, median (IQR)	3 (1,7)	2 (1,4)	Sionean *et al.*[15]
Relationship duration (quantiles)	2.0 days (1.0-4.5), 11.0 days (9.5-12.5), 1.3 months (1.1-1.6), 4.9 months (3.8-6.3), 11.9 months (10.0-14.9) [a]		Burington *et al.*[17]
Mean number of vaginal sex acts per month (95% CI)	4.9 (4.3, 5.5)	4.9 (4.3, 5.6)	Leichliter *et al.*[16]
Median number of vaginal sex acts per month (95% CI)	2.5 (1.9, 3.0)	1.9 (1.5, 2.3)	Leichliter *et al.*[16]
Probability of unprotected sex with main partner (relationship duration ≥ 1 month)	90.5%		Sionean *et al.*[15]
Probability of unprotected sex with main partner (relationship duration <1 month)	72.5%		Sionean *et al.*[15]

Abbreviations: IQR- interquartile range, CI- confidence interval
[a] Quantiles with confidence intervals (2.5^{th} and 97.5^{th} percentiles the bootstrap distribution based on 2000 bootstrap samples).

Following the partnering algorithm, HIV-diagnosed agents are eligible to receive highly active antiretroviral therapy (HAART) and HIV-undiagnosed agents are eligible to undergo HIV testing. HIV-infected agents on HAART also have specific probability of discontinuation of care, which corresponds to ceasing HAART and experiencing viral rebound. The probability of receiving HAART was calibrated to historical trends in rates of viral suppression observed in Philadelphia from 2005-2015 using HIV surveillance data

received from the AIDS Activities Coordinating Office (AACO). Specifically, increased access to HAART during this time period increased the percentage of HIV-diagnosed individuals achieving viral suppression from 4% in 2005 to over 50% by 2015. A probability function was created which reflected this non-linear growth in HAART coverage such that the likelihood of enrolling in ART treatment was based on the simulation time step.

Agents that initiate HAART are assigned an adherence value, *A*, in the time step following initiation. HIV surveillance data from AACO was used to parameterize the proportion of individuals initiating HAART who achieved ≥90% adherence (*A* ≥90%). Agents that do not achieve ≥90% adherence were assigned to one of four other adherence quartiles (0-29%, 30-49%, 50-69%, 70-89%) with equal probability. We assumed that adherence is constant while an agent is on therapy. We also note that, in this model, we do not account for type of HAART regimen or the development of virologic resistance; as such, the effect of adherence on virologic suppression and subsequent risk of transmission represent mean values observed in the treated population. HIV transmission risk by HAART adherence level is discussed in detail within the section titled "HIV Transmission".

All agents who were not previously diagnosed with HIV were eligible to undergo HIV testing. The probability of HIV testing was determined using a probability function that resulted in an average of 3.43% of male agents and 3.93% of female agents without a known HIV diagnosis being tested monthly, held constant throughout the model runs. HIV testing parameterization was specific to Philadelphia and was drawn from NHBS data.[14] In addition to the assumption that the likelihood of HIV testing was constant over

time, we also assumed that HIV testing had 100% sensitivity and specificity. Based on national estimates, our model assumed that 75% of HIV-infected individuals were diagnosed.[9]

The discontinuation of HAART was parameterized using several national estimates. African Americans and women have been shown to have particularly high hazards of HAART discontinuation.[18,19] In a longitudinal study of 753 HIV-infected men and women (50% African American), Robison et al. estimated that 61% (n=298/492) of women and 59% (n=534/913) of African Americans discontinued HAART within twelve months of initiation.[18] Another longitudinal study of HIV-infected women within the Women's Interagency HIV Study estimated that 25% of women discontinued HAART for at least six months during the study's five year follow-up. Within our model, HIV-diagnosed male agents on HAART had 42% probability of discontinuing HAART per year while female agents on HAART had a 52% probability per year.[18,19] Agents who discontinue therapy at time step j re-initiate care at any time $t > j$ at the same rate as those who are newly diagnosed. Parameter estimates related to HIV screening and treatment are presented in **Table S3**.

Table S3. Parameter estimates related to HIV screening and treatment.

Variable	Base estimate		Data Source
	Male Agents	*Female Agents*	
ART coverage and initiation	Varies over time		Calibrated, AACO
HIV testing (monthly %)	3.43%	3.93%	NHBS[14]
Proportion of HIV-infected individuals with HIV diagnosis	75%	75%	Marks *et al.*[9]
Proportion of HIV-infected individuals on ART achieving viral suppression	Varies over time		AACO
Discontinuation of HAART (% per year)	42%	52%	Robison *et al.*[18], Adieh-Grant *et al.*[19]

Initialization

At model initialization (i.e., at time *t*=0 of a simulation run), there were 110,000 agents, roughly a quarter of our target population of 440,403. Runs were made with a quarter of the target population due to computing constraints. State variables were set stochastically from probability functions and were allowed to vary among simulations. Initial values were based on the published literature and HIV surveillance data from the city of Philadelphia. The model was initialized with an HIV prevalence of 1.58% for men and 1.18% for women based on 2005 HIV surveillance data for heterosexual African American men and women living in Philadelphia. Surveillance data from 2005 on the proportion of HIV-infected individuals with lab results indicating viral suppression and CD4 lab results indicating AIDS were used to parameterize the proportion of HIV-diagnosed individuals on HAART and AIDS prevalence. HIV prevalence within correctional facilities was based on an estimate from a study of Philadelphia HIV testing within urban jails from 2008 to 2009.[20] The estimate for AIDS prevalence within correctional facilities was based on national data for the northeastern United States from 2009-2010.[21] Race-specific or Philadelphia-specific data on AIDS prevalence in correctional facilities were not available. Initial conditions are presented are presented in **Table S4.**

Table S4. Initial model conditions.

Variable	Base estimate		Data Source
	Male Agents	*Female Agents*	
Community size (*N*)- ¼ target population	44,500	65,571	Calculated, U.S. Census 2000, Lieb *et al.*[8]
HIV prevalence (%)	1.58%	1.18%	Calculated, AACO
Proportion of HIV-infected individuals with HIV diagnosis	75%	75%	Marks *et al.*[9]
Proportion of HIV-diagnosed individuals on HAART (%)	3.75%	4.05%	Assumed/calibrated, AACO
AIDS prevalence	67.8%	57.3%	Calculated, AACO
Proportion incarcerated (%)	2.74%	n/a	Estimated, Goldkamp *et al.*[10]
HIV prevalence in correctional facilities (%)	3.5%	n/a	Beckwith *et al.*[20]
AIDS prevalence in correctional facilities (%)	18.94%	n/a	Maruschak *et al.*[21]

HIV Disease Progression and Mortality

A detailed description of our HIV disease progression model has been published previously.[3] Following acute HIV infection, which lasts for 3 monthly time steps, based on previous data [22], HIV-infected agents with latent stage infection progress to AIDS at a rate dependent on treatment enrollment status and adherence.[23-25] This approach assures that there will be a large variation in time-to-AIDS for the HIV-infected agent population, but also has a notable limitation in that all agents may progress to AIDS with equal probability at each point following acute infection, meaning that a very small portion may progress to AIDS sooner than population-level estimate and clinical case-studies

suggest.[26] However, these instances of early progression are very rare. The probability of progression to AIDS for each adherence category is listed in **Table S5**.

Table S5. Parameters and data sources for HIV disease progression and mortality.

Variable	Base Estimate		Source
	Male Agents	*Female Agents*	
**Progression to AIDS (annual probability)[a]**			Egger *et al.*[23], Moss *et al.*[24], Porter *et al.*[25]
Not on ART	0.005		
0% – 29% adherent to ART	0.005		
30% – 49% adherent to ART	0.0039		
50% – 69% adherent to ART	0.0032		
70% – 89% adherent to ART	0.0025		
≥90% adherent to ART	0.0008		
**All-Cause Mortality Rate (per 1,000 person-years)**			
Among HIV negative agents	7.31	3.77	NCHS[27]
Among HIV positive agents, not on ART	16.5	16.5	Estimated: Siddiqi *et al.*[28]
Among HIV positive agents, on ART	7.31	3.77	NCHS[27], Siddiqi *et al.*[28]
Among Agents diagnosed with AIDS	33	33	Estimated: Siddiqi *et al.*[28]

Abbreviations: HIV – human immunodeficiency virus; HAART –highly active antiretroviral therapy, NCHS – National Center for Health Statistics.
[a] HIV surveillance data used to estimate the proportion of agents achieve ≥90% of adherence upon initiating HAART (the remaining proportion are assigned to four other quartiles [0% - 29%, 30% - 49%, 50% - 69%, 70% - 89%] with equal probability)

There is a baseline probability of all-cause mortality for each agent class, as well as an increased probability of all-cause mortality for HIV-infected agents, based on their AIDS status and HAART adherence; these values are also presented in **Table S5**. All-cause mortality for HIV-uninfected agents was calculated using age standardized mortality rates reported from the National Center for Health Statistics (CDC WONDER Online Database) for African American men and women aged 15-64 averaged from 2005-

2014.[27] HIV-infected agents on HAART were assumed to have the same average mortality rate as HIV-uninfected agents based on the published literature.[29] Mortality rates for African-American HIV-infected men and women not on HAART and diagnosed with AIDS were estimated based on a national study.[28] The model does not directly estimate mortality as a result of HIV infection or AIDS, rather it assigns unique values for all-cause mortality based on disease and treatment status.

HIV Transmission

To model sexual HIV transmission, we simulated the monthly number of sexual acts and the proportion of those acts that are unprotected between each pair of connected, serodiscordant agents, as described above. Specifically, at every time step, the number of sex acts for each agent was drawn from a Poisson distribution, with means shown in **Table S2**. We assumed that HIV-diagnosed agents were 50% less likely to engage in unprotected intercourse with their partners, based on prior literature.[30] We assumed that undiagnosed, HIV-infected agents engaged in sexual risk behavior at the same probability as HIV negative individuals.

If a pair of serodiscordant agents engage in sexual risk behavior, the model implements a stochastic algorithm to determine whether HIV transmission occurs. To calculate the probability of HIV transmission to an uninfected partner, we use per-act probabilities (β_a) shown in **Table S6**, based on previously published estimates.[31,32] We did not model unprotected anal intercourse between dyads.

The probabilities listed in **Table S6** represent the average risk of transmission during an unprotected vaginal sexual intercourse event during latent stage HIV infection. Viral load is modeled implicitly, such that these values represent a mean set-point viral

load (approximately 4 $\log_{10}$ copies/mL) in the HIV-infected population.[33] To account for higher viral load and an increased risk of transmission during acute phase infection, we multiplied these probabilities by a factor of 4.3 during the first three time steps following seroconversion, which represents the average increase in transmission risk during acute HIV infection.[34,35]

In order to calculate the overall transmission risk per partnership per time step, β_p, we employed a Binomial process model,[36] i.e.:

$$\beta_p \sim Bin(n, \beta_a) = \frac{\beta_p!}{(\beta_p - n)!\,n!} {\beta_a}^{\beta_p} (1-\beta_a)^{n-\beta_p}, \qquad \beta_p \epsilon \{1, \cdots, n\}$$

where β_a is the per-act transmission probability. As shown in **Table S6** and as described below, these probabilities were also dependent on the HIV treatment status and adherence pattern of the HIV-infected partner. We modeled the relationship between HAART adherence and the suppression of viral replication implicitly, such that, for each adherence value (*A*), we assigned a different value for per-contact risk of HIV transmission $\beta_{a,A}$. As shown in **Table S6**, higher values of HAART adherence reduce the per-event probability of HIV transmission. These values have been estimated from previously conducted studies investigating the relationship between adherence and viral load,[37] as well as the effect of viral suppression on HIV transmission.[32] The number of "trials" (total number of unprotected sex events) per partnership per time step, *n*, was determined for each dyad as described above.

Table S6. Parameters and data sources for HIV transmission.

Variable	Base Estimate		Source
	Male Agents	*Female Agents*	
HIV transmission risk per unprotected vaginal sex act (chronic phase) by adherence to HAART level			Gray *et al*.[31], Quinn *et al*.[32]
Not on HAART	0.0010		
0-29% adherent	0.0010		
30-49% adherent	0.0008		
50-69% adherent	0.0004		
70-89% adherent	0.0002		
≥90% adherent	0.0001		
Increase in infectivity during acute stage infection	4.3		Bellan *et al*.[35], Wawer *et al*.[34]
Early phase duration (months)	3		Bellan *et al*.[35], Wawer *et al*.[34]

Incarceration

Incarceration was implemented according to the following processes and parameters. We defined incarceration inclusively as being held in a prison or any other kind of detention facility for at least a month or longer. We were not able to account for short-term (i.e., <1 month) jail stays in this model. This definition is consistent with previously published agent-based modeling analyses of incarceration.[38,39] As such, we assumed that the effect of incarceration on HIV treatment outcomes and agent behavioral processes does not vary across correctional environments. In other words, the parameter values shown in **Table S7** reflect average incarceration experiences.

Table S7. Parameters and data sources for the impact of incarceration.

Variable	Base estimate		Data Source
	Male Agents (African American)	*Female Agents*	
Proportion currently incarceration (%)	2.7-2.8%	n/a	Calculated: Goldkamp *et al.*[10], Mauer *et al.*[12], Sakala *et al.*[40]
Annual rate of incarceration in jail per 100,000 people for first-time offenders	100	n/a	PCS[11]
Annual rate of incarceration in jail per 100,000 people for those with a prior record	276	n/a	PCS[11]
Average length of minimum jail sentence (months)	8	n/a	PCS[11]
Average length of maximum jail sentence (months)	21.6	n/a	PCS[11]
Annual rate of incarceration in prison per 100,000 for first-time offenders	75	n/a	PCS[11]
Annual rate of incarceration in prison per 100,000 for those with a prior record	251	n/a	PCS[11]
Average length of minimum prison sentence (months)	45.6	n/a	PCS[11]
Average length of maximum jail sentence (months)	96	n/a	PCS[11]
HIV testing in correctional facility (probability upon intake)	69%	n/a	Beckwith *et al.*[20]
Percent of HIV-infected inmates on HAART while incarcerated	40%	n/a	Iroh *et al.*[41]
Mean number of sex partners for high risk groups[a]	1.8 monthly	3.25 by month 10	Cooper *et al.*[42]
Percent of HIV-infected inmates maintained on HAART at six months post-release	21%	n/a	Iroh *et al.*[41]
Probability of HAART re-initiation for those who discontinued post-release	0.75 community probability	n/a	Estimated: Iroh *et al.*[41]

Abbreviations: n/a- not applicable; PCS- Philadelphia Commission on Sentencing
[a] All male agents released from prison or jail are high-risk for six months following release. 30% of women with a main partner who is incarcerated are high-risk immediately upon a partner's incarceration and 50% are high-risk if the relationship dissolves during a partner's incarceration.

At model initialization, the proportion of agents assigned to incarceration was based on previously published estimates. In 2006, the Crime and Justice Research Center at Temple University submitted a report to the City of Philadelphia characterizing the demographic makeup of incarcerated individuals within the Philadelphia Department of Corrections.[10] At the end of November 2005, there were 8,541 individuals confined within the Philadelphia Prison system either serving out sentences, awaiting bail or sentencing, detained on probation or parole violation, held on a bench warrant or held for another reason. We excluded 300 prisoners who were transferees from Delaware Country (n=8,241). Approximately 90% of inmates were men and 73% were African American, resulting in approximately 5,414 African American male prisoners. Using this count as the numerator and the U.S. Census 2000 count for the number of African American men over the age of 18 as the denominator (5,414/191,525), we estimated that between 2.7-2.8% of Philadelphia's African American male population was currently incarcerated at model initialization. This proportion was similar to other reported estimates (2.79% in 2005, 3.27% in 2010) for African American men from the state of Pennsylvania.[12,40]

A 2007 report from the Urban League of Philadelphia reported detailed information on sentence lengths and incarceration rates per 100,000 people for African American and white men in Philadelphia using 2006 data from the Philadelphia Commission on Sentencing.[11] Rates of incarceration and sentence lengths were reported by type of correctional facility (jail vs. prison) and recidivism status (first-time offender vs. prior incarceration). For example, the annual rate of incarceration in prison per 100,000 people for those with a prior record was 251 per 100,000 for African American men. These rates

and sentence lengths were used to parameterize incarceration within the model and held constant through model runs (i.e., we modeled a constant rate of incarceration as race-specific rates and sentence lengths were not available for each year within the study period).

When an agent became incarcerated, we assumed that all sexual contact with other agents temporarily ceased. Thus, while an HIV-infected agent is incarcerated, HIV transmission cannot occur. Since our focus was on community HIV incidence, we did not model HIV transmission between incarcerated agents. Main relationships (≥1 month in duration) can be maintained during incarceration, but have a 55% probability of dissolving during incarceration, based on previous research.[43] If the relationship is maintained during incarceration, the number of vaginal sexual intercourse acts per month is set to 0 (i.e., sex during conjugal visits was not modeled). Based on previously published research on HIV testing within Philadelphia correctional facilities and a systematic review on the HIV care continuum, we assumed that 69% of agents were tested for HIV at intake throughout the study period.[20] During incarceration, 40% of diagnosed HIV-infected men were assumed to achieve viral suppression.[41] These parameters were held constant throughout the study period.

Within the model, incarceration was programmed to directly impact sexual risk behavior and HIV care engagement. These behaviors were parameterized using observational studies on the impact of incarceration and partner incarceration.[41,42,44-46] Women were only eligible to experience increased risk behavior if the incarcerated partner was a main partner (i.e., relationship ≥1 month). For the primary analysis, the average duration of risk behavior for women was either six months following a

relationship's dissolution or throughout a partner's incarceration, whichever was applicable. Within this model, 30% of women with incarcerated partners initiated high-risk behavior immediately upon the partner's incarceration. In addition, if a relationship dissolved during a partner's incarceration, a female agent had a 50% probability of entering the high-risk group. The remaining women maintained the same risk profile they had before a partner's incarceration. Limited information was available on the proportion of women with incarcerated partners who increase sexual risk behaviors or the average duration of high-risk behavior. Therefore, both of these parameters were varied in sensitivity analyses. For women, high-risk behavior related to partner incarceration consisted of increasing the number of sexual partners from a median of 2 (interquartile range [IQR]: 1, 4) per year to an average of 3.25 (standard deviation=3.25) by ten months, based on an observational study of women with recently incarcerated partners.[42]

High-risk behavior related to incarceration for male agents initiated upon release from prison or jail. Based on a set of studies published on Project START, an HIV-prevention program focused on reducing HIV/STI risk in young men following release from prison, the simulation modeled an increase in the number of sexual partners during the six months post-release from a median of 3 (IQR: 1,7) per year to an average of 1.8 per month.[45,46] All male agents who were released from prison or jail underwent this high-risk period. In addition, HIV-infected men were less likely to be retained in HIV care post-release.[47] A recent systematic review of U.S. data found that 40% percent of prisoners are on HAART while incarcerated, while only 21% remain on HAART after release.[41] In our model, we set the probability of initiating HAART (for agents newly diagnosed at entry) such that overall treatment coverage was 40% in the correctional environment. Agents

already on HAART and those newly diagnosed at entry may discontinue therapy upon release. Specifically, in the model, the probability of discontinuing HAART after release was estimated to be $1 - 0.21/0.40 = 0.475$ by six months post-release. Once an agent with a history of incarceration discontinued HAART, the probability of re-initiating HAART was assumed to be 75% that of the community probability. This specific parameter was not available through the existing literature, but reflects previous research that has found lower rates of viral suppression after release compared to before incarceration.[41,48]

Once an incarcerated agent has served his sentence length, he is returned to the eligible pool of agents and seeks to re-establish links with partner(s). First, the previously incarcerated agent re-forms links with partner(s) he had prior to incarceration. To do so, the simulation takes a snapshot of the existing relationships at the time of incarceration and stores this information. In the next time step following release, the model compares the number of partners the formerly incarcerated agent's partner(s) has with the drawn value(s) for $j_{i,t}$. If the current number exceeds $j_{i,t}$, partnerships are dissolved at random until that number is obtained. If the formerly incarcerated agent is dropped from that partner's network, the formerly incarcerated agent becomes eligible to form new partnerships. Thus, agents returning to the "community" may either resume old relationships or establish new relationships.[49]

In summary, incarceration in the model directly affects sexual networks through the disruption of existing partnerships and acquisition of new ones, which been demonstrated in prior studies, and is thought to play an important role in perpetuating HIV transmission in communities with high rates of incarceration.[50-52] Increased rates of partner concurrency and relationship turnover emerged as result of these changed

behaviors rather than as a result of programmed parameters. In addition, incarceration negatively impacted HIV care engagement for HIV-infected men and increased the likelihood of HIV transmission in the post-release period. Parameters and data sources related to incarceration are presented in **Table S7**.

Status Quo Scenario and Model Scenarios

The model and parameters described above and summarized in Tables S1-S7 were used to generate the "*status quo*" model. Specifically, outputs from the *status quo* model represent HIV incidence and HIV prevalence estimates (per 100,000 persons) reflecting current, "real world" epidemic dynamics seen in Philadelphia from 2005-2015. In the accompanying manuscript, we describe the average HIV incidence and number of HIV transmissions for a 10-year period. Given the stochasticity inherent in these models, the *status quo* case was repeated in Monte Carlo simulations 200 times with a quarter of the total population size (n=110,000) in order to obtain stable point estimates.

We then constructed a counterfactual scenario where no agent experienced incarceration. Six scenarios varying key parameters hypothesized to influence the impact of mass incarceration were then compared to this counterfactual scenario. Specifically, we varied the duration of high-risk behavior for men post-release (3 months, 24 months), duration of high-risk behavior for women (3 months, 24 months), the proportion of women engaging in high-risk behavior (0%, 100%), HAART coverage for HIV-infected men at six months post-release (0%, 100%), the relative risk of incarceration for HIV-infected men compared to HIV-uninfected men (2, 5), and doubled the risk of HIV transmission per unprotected vaginal sex act to account for the increased probability of a current STI among high-risk agents (for high-risk period only, for the

remainder of the model run). These parameters are summarized in **Table S8.** In order to isolate the effect of the selected parameter, we held values for the other key parameters constant at a referent value. Each scenario was run 100 times with ¼ the total population size (n=110,000) using Markov Chain Monte Carlo simulations; results were then scaled to reflect the target population (n=440,000). The average number and percentage of HIV infections averted among African American women were calculated and compared.

Table S8. Parameters to evaluate the potential drivers of the impact of incarceration.

**Variable**	**Estimate**		
	Lower Bound	*Upper Bound*	*Base*
Duration of high risk behavior for male agents (months)	3	24	6
Duration of high risk behavior for female agents (months)	3	24	6 or length of partner's incarceration
Proportion of women engaging in high risk behavior	0%	100%	Varies
Percent of HIV-infected inmates maintained on HAART at six months post-release	0%	100%	21%
Relative risk of incarceration for HIV-infected men vs. HIV-uninfected men	2	5	1
Doubling of HIV acquisition risk per unprotected vaginal sex act for high-risk individuals	High-risk period only	For agent's lifetime	Base estimate

Model Calibration

To calibrate the model, we employed an iterative indirect approach, following previously published recommendations.[53] First, the set of empirical behavioral and risk parameters were applied to the model agents, and preliminary outputs (e.g., HIV incidence among specific groups, agent class distributions) were assessed and compared to historic datasets. Sweeping sensitivity analysis was performed to determine the model stability regarding key parameters, including the distribution of agent classes, HIV prevalence, HIV incidence, and incarceration prevalence over the simulation run time. These stress tests allowed us to measure qualitative and quantitative effects of core parameters, and through this we determined input variables that held the most significant changes in model outputs.

Model refinement was then conducted by adjusting key parameters for which there existed greater uncertainty in their values (e.g., monthly risk of incarceration per month per agent classification) to minimize differences between model output and key historic datasets. Specifically, we focused on fitting the data to multiple outputs, including HIV incidence by agent class (e.g., gender), HIV prevalence by agent class, and HIV diagnosis rates per 100,000 population per year. We then ran a series of revised simulations and continued this process iteratively, until each set of model output approximated the historic data. Although this process does not necessarily guarantee model validity, it does permit the exclusion of parameter values that do not adequately reproduce the empiric data.[53] Well documented and previously published calibrated outputs for other variables of interest (e.g., HIV disease progression rates) remained unchanged.[54,55]

Technical Details

The model was coded, tested, and calibrated in an open-source programming language (Python™ version 2.7.2). The simulation generated an agent matrix of 110,000 agents of varying classifications and substrata, which were managed by independent Python dictionaries. At each time step, information on the current agent state and each agent's partners were recorded, agents were assigned partners using the methods described above, and then interacted with each other along their network edges. All agents performed their acts simultaneously during a time step, requiring careful consideration to the order of operations and transition of states of each agent at this time. Agents then engaged in HIV treatment and were incarcerated, followed by a "die and replace" algorithm. This process was continued iteratively until the desired simulation time was met.

The program was run on a Beowulf supercomputing cluster consisting of multiple computer nodes and one head node, each with quad-core Intel™ CPUs and at least 8 GB of RAM. The base case and counterfactual models were run for a duration of 156 time steps (13 years). The first 36 time steps (3 years) were omitted from final results to as this period was necessary in order to reach a steady-state and accurately reflect historical trends in empirical data for the status quo model. Results from the final 120 time steps (10 years) averaged over a total of 100 unique runs, each with a stochastically generated population following the parameters provided in Table S1-S8. Average runtime for a complete single iteration of the model was approximately 45 minutes.

References

1. Grimm V, Berger U, Bastiansen F, et al. A standard protocol for describing individual-based and agent-based models. *Ecological Modelling.* 2006;198(1–2):115-126.
2. Grimm V, Berger U, DeAngelis DL, Polhill JG, Giske J, Railsback SF. The ODD protocol: A review and first update. *Ecological Modelling.* 2010;221(23):2760-2768.
3. Marshall BD, Paczkowski MM, Seemann L, et al. A complex systems approach to evaluate HIV prevention in metropolitan areas: preliminary implications for combination intervention strategies. *PLoS One.* 2012;7(9):e44833.
4. Monteiro JF, Galea S, Flanigan T, Monteiro Mde L, Friedman SR, Marshall BD. Evaluating HIV prevention strategies for populations in key affected groups: the example of Cabo Verde. *Int J Public Health.* 2015;60(4):457-466.
5. Monteiro JF, Escudero DJ, Weinreb C, et al. Understanding the effects of different HIV transmission models in individual-based microsimulation of HIV epidemic dynamics in people who inject drugs. *Epidemiol Infect.* 2016;144(8):1683-1700.
6. Escudero DJ, Lurie MN, Mayer KH, et al. Acute HIV infection transmission among people who inject drugs in a mature epidemic setting. *AIDS.* 2016;30(16):2537-2544.
7. Marshall BD, Friedman SR, Monteiro JF, et al. Prevention and treatment produced large decreases in HIV incidence in a model of people who inject drugs. *Health Aff (Millwood).* 2014;33(3):401-409.
8. Lieb S, Fallon SJ, Friedman SR, et al. Statewide Estimation of Racial/Ethnic Populations of Men Who Have Sex with Men in the U.S. *Public Health Reports.* 2011;126(1):60-72.
9. Marks G, Crepaz N, Janssen RS. Estimating sexual transmission of HIV from persons aware and unaware that they are infected with the virus in the USA. *AIDS.* 2006;20(10):1447-1450.

10. Goldkamp JS, Vîlcică E, Weiland D, Ke W. *Confinement and the justice process in Philadelphia: Its features and implications for planning.* Temple University;2006.

11. Urban League of Philadelphia. *The State of Black Philadelphia.* Urban League of Philadelphia;2007.

12. Mauer M, King RS. *Uneven justice: State rates of incarceration by race and ethnicity.* Sentencing Project Washington, DC; 2007.

13. Hamilton DT, Handcock MS, Morris M. Degree distributions in sexual networks: a framework for evaluating evidence. *Sex Transm Dis.* 2008;35(1):30-40.

14. Centers for Disease Control and Prevention. *HIV Infection, Risk, Prevention, and Testing Behaviors among Heterosexuals at Increased Risk of HIV Infection—National HIV Behavioral Surveillance, 20 U.S. Cities, 2013.* 2015.

15. Sionean C, Le BC, Hageman K, et al. HIV Risk, prevention, and testing behaviors among heterosexuals at increased risk for HIV infection--National HIV Behavioral Surveillance System, 21 U.S. cities, 2010. *MMWR Surveill Summ.* 2014;63(14):1-39.

16. Leichliter JS, Chesson HW, Sternberg M, Aral SO. The concentration of sexual behaviours in the USA: a closer examination of subpopulations. *Sex Transm Infect.* 2010;86 Suppl 3:iii45-51.

17. Burington B, Hughes JP, Whittington WL, et al. Estimating duration in partnership studies: issues, methods and examples. *Sex Transm Infect.* 2010;86(2):84-89.

18. Robison LS, Westfall AO, Mugavero MJ, et al. Short-term discontinuation of HAART regimens more common in vulnerable patient populations. *AIDS Res Hum Retroviruses.* 2008;24(11):1347-1355.

19. Ahdieh-Grant L, Tarwater PM, Schneider MF, et al. Factors and temporal trends associated with highly active antiretroviral therapy discontinuation in the Women's Interagency HIV Study. *J Acquir Immune Defic Syndr.* 2005;38(4):500-503.

20. Beckwith CG, Nunn A, Baucom S, et al. Rapid HIV testing in large urban jails. *American journal of public health.* 2012;102(S2):S184-S186.

21. Maruschak L. HIV in Prisons, 2001–2010. Bureau of Justice Statistics, US Department of Justice. 2012.

22. Hollingsworth TD, Anderson RM, Fraser C. HIV-1 transmission, by stage of infection. *J Infect Dis.* 2008;198(5):687-693.

23. Egger M, May M, Chene G, et al. Prognosis of HIV-1-infected patients starting highly active antiretroviral therapy: a collaborative analysis of prospective studies. *Lancet.* 2002;360(9327):119-129.

24. Moss AR, Bacchetti P. Natural history of HIV infection. *AIDS.* 1989;3(2):55-62.

25. Porter K, Babiker A, Bhaskaran K, et al. Determinants of survival following HIV-1 seroconversion after the introduction of HAART. *Lancet.* 2003;362(9392):1267-1274.

26. Gomez G, Lagakos SW. Estimation of the infection time and latency distribution of AIDS with doubly censored data. *Biometrics.* 1994;50(1):204-212.

27. Multiple Cause of Death 1999-2014 CDC WONDER Online Database; 2015. http://wonder.cdc.gov/mcd-icd10.html Accessed 11/18/2016.

28. Siddiqi AE, Hu X, Hall HI. Mortality among blacks or African Americans with HIV infection--United States, 2008-2012. *MMWR Morb Mortal Wkly Rep.* 2015;64(4):81-86.

29. !!! INVALID CITATION !!! 27,28.

30. Marks G, Crepaz N, Senterfitt JW, Janssen RS. Meta-analysis of high-risk sexual behavior in persons aware and unaware they are infected with HIV in the United States: implications for HIV prevention programs. *J Acquir Immune Defic Syndr.* 2005;39(4):446-453.

31. Gray RH, Wawer MJ, Brookmeyer R, et al. Probability of HIV-1 transmission per coital act in monogamous, heterosexual, HIV-1-discordant couples in Rakai, Uganda. *Lancet.* 2001;357(9263):1149-1153.

32. Quinn TC, Wawer MJ, Sewankambo N, et al. Viral load and heterosexual transmission of human immunodeficiency virus type 1. Rakai Project Study Group. *N Engl J Med.* 2000;342(13):921-929.

33. Little SJ, McLean AR, Spina CA, Richman DD, Havlir DV. Viral dynamics of acute HIV-1 infection. *J Exp Med.* 1999;190(6):841-850.

34. Wawer MJ, Gray RH, Sewankambo NK, et al. Rates of HIV-1 transmission per coital act, by stage of HIV-1 infection, in Rakai, Uganda. *J Infect Dis.* 2005;191(9):1403-1409.

35. Bellan SE, Dushoff J, Galvani AP, Meyers LA. Reassessment of HIV-1 acute phase infectivity: Accounting for heterogeneity and study design with simulated cohorts. *PLoS Med.* 2015;12(3):e1001801.

36. Kaplan EH. Modeling HIV infectivity: must sex acts be counted? *J Acquir Immune Defic Syndr.* 1990;3(1):55-61.

37. Bangsberg DR, Hecht FM, Charlebois ED, et al. Adherence to protease inhibitors, HIV-1 viral load, and development of drug resistance in an indigent population. *AIDS.* 2000;14(4):357-366.

38. Lum K, Swarup S, Eubank S, Hawdon J. The contagious nature of imprisonment: an agent-based model to explain racial disparities in incarceration rates. *J R Soc Interface.* 2014;11(98):20140409.

39. Knittel AK, Snow RC, Riolo RL, Griffith DM, Morenoff J. Modeling the community-level effects of male incarceration on the sexual partnerships of men and women. *Soc Sci Med.* 2015;147:270-279.

40. Sakala L. *Breaking down mass incarceration in the 2010 census: State-by-state incarceration rates by race/ethnicity.* 2014.

41. Iroh PA, Mayo H, Nijhawan AE. The HIV care cascade before, during, and after incarceration: a systematic review and data synthesis. *American journal of public health.* 2015;105(7):e5-e16.

42. Cooper HL, Caruso B, Barham T, et al. Partner incarceration and African-American women’s sexual relationships and risk: A longitudinal qualitative study. *Journal of Urban Health.* 2015;92(3):527-547.

43. Khan MR, Behrend L, Adimora AA, Weir SS, Tisdale C, Wohl DA. Dissolution of primary intimate relationships during incarceration and associations with post-release STI/HIV risk behavior in a Southeastern city. *Sex Transm Dis.* 2011;38(1):43-47.

44. Adimora AA, Schoenbach VJ, Martinson F, Donaldson KH, Stancil TR, Fullilove RE. Concurrent sexual partnerships among African Americans in the rural south. *Ann Epidemiol.* 2004;14(3):155-160.

45. Morrow KM, Project SSG. HIV, STD, and hepatitis risk behaviors of young men before and after incarceration. *AIDS Care.* 2009;21(2):235-243.

46. Seal D, Eldrige G, Kacanek D, Binson D, MacGowan R, Group PSS. A longitudinal, qualitative analysis of the context of substance use and sexual behavior among 18-to 29-year-old men after their release from prison. *Social science & medicine.* 2007;65(11):2394-2406.

47. Iroh PA, Mayo H, Nijhawan AE. The HIV Care Cascade Before, During, and After Incarceration: A Systematic Review and Data Synthesis. *Am J Public Health.* 2015:e1-e12.

48. Meyer JP, Cepeda J, Springer SA, Wu J, Trestman RL, Altice FL. HIV in people reincarcerated in Connecticut prisons and jails: an observational cohort study. *Lancet HIV.* 2014;1(2):e77-e84.

49. Adimora AA, Schoenbach VJ. Social context, sexual networks, and racial disparities in rates of sexually transmitted infections. *J Infect Dis.* 2005;191(Suppl 1):S115-S122.

50. Adimora AA, Schoenbach VJ. Social context, sexual networks, and racial disparities in rates of sexually transmitted infections. *J Infect Dis.* 2005;191 Suppl 1:S115-122.

51. Khan MR, Miller WC, Schoenbach VJ, et al. Timing and duration of incarceration and high-risk sexual partnerships among African Americans in North Carolina. *Ann Epidemiol.* 2008;18(5):403-410.

52. Khan MR, Wohl DA, Weir SS, et al. Incarceration and risky sexual partnerships in a southern US city. *J Urban Health.* 2008;85(1):100-113.

53. Windrum P, Fagiolo G, Moneta A. Empirical validation of agent-based models: alternatives and prospects. *J Artif Soc Soc Simul.* 2007;10(2):8.

54. Marshall BDL, Friedman SR, Monteiro JF, et al. Prevention and treatment produced large decreases in HIV incidence in a model of people who inject drugs. *Health Aff (Millwood).* 2014;33(3):401-409.

55. Marshall BDL, Paczkowski MM, Seemann L, et al. A complex systems approach to evaluate HIV prevention in metropolitan areas: Preliminary implications for combination intervention strategies. *PLoS One.* 2012;7(9):e44833.
